# Evaluation of the SAMEO-ATO surgical classification in a Dutch cohort

**DOI:** 10.1007/s00405-020-06109-1

**Published:** 2020-06-11

**Authors:** Fleur A. ten Tije, Sietze Alkema, Lisa van der Putten, Jan Pieter Koopman, Joeri Buwalda, Sophia E. Kramer, Robert Jan Pauw, Paul Merkus, Dutch Cholesteatoma Data Consortium

**Affiliations:** 1 Department of Otorhinolaryngology-Head and Neck Surgery, Section Ear and Hearing, Amsterdam University Medical Centers, Amsterdam Public Health Research Institute, Vrije Universiteit Amsterdam, De Boelelaan, VUmc, P.O. box 7057, 1007 MB Amsterdam, The Netherlands; 2 Department of Otorhinolaryngology-Head and Neck Surgery, Noordwest Ziekenhuisgroep, Alkmaar, Den Helder, The Netherlands; 3 Department of Otorhinolaryngology-Head and Neck Surgery, Haga Ziekenhuis, The Hague, The Netherlands; 4 Department of Otorhinolaryngology-Head and Neck Surgery, Deventer Ziekenhuis, Deventer, The Netherlands; 5 Department of Otorhinolaryngology-Head and Neck Surgery, Erasmus University Medical Center, Rotterdam, The Netherlands

**Keywords:** Cholesteatoma, Surgical procedures, Classification, Registration

## Abstract

**Purpose:**

Differences in the definition and classification of cholesteatoma hinders comparing of surgical outcomes of cholesteatoma. Uniform registration is necessary to allow investigators to share and compare their findings. For many years surgical cholesteatoma procedures were divided into two main groups: canal wall up mastoidectomy (CWU) and canal wall down mastoidectomy (CWD). Recently, mastoid obliteration can be added to both procedures. Because of great variation within these main groups, the International Otology Outcome Group (IOOG) proposed the new SAMEO-ATO classification system to categorize tympanomastoid operations. The aim of our study was to correlate the mastoid bone extirpation (M-stage) with the contemporary (CWU, CWD with or without obliteration) system.

**Methods:**

Demographic characteristics and type of performed surgery were registered for 135 cholesteatoma patients from sixteen hospitals, both secondary and tertiary care institutions, across the Netherlands. In addition, the surgical reports were collected, retrospectively classified according to the contemporary system and the new system and compared. Correlations of the outcomes were calculated.

**Results:**

In total, there were 112 CWU and 14 CWD (both with or without obliteration) suitable for correlation analysis. *Z* test for correlation between the M-stage and CWU procedure was significant for M1a and M1b procedure and significant for M2c with the CWD procedure.

**Conclusion:**

The newly proposed SAMEO-ATO classification seems to be more detailed in the registration of surgical procedures than surgeons currently are used to. All M-stages of the SAMEO-ATO system are correlating well to the standard CWU and CWD except one ‘in between’ M-stage.

**Electronic supplementary material:**

The online version of this article (10.1007/s00405-020-06109-1) contains supplementary material, which is available to authorized users.

## Introduction

The management of cholesteatoma remains a challenge for ENT surgeons around the world. Unfortunately, due to differences in the classification, reporting and management of cholesteatoma by professionals worldwide, it is difficult to compare the results of cholesteatoma treatment as reported in literature [1]. A classification system making uniform registration feasible is necessary to allow investigators to share their findings and make comparison of outcomes across different surgical techniques possible [2]. The outcome of the treatment of cholesteatoma depends mostly on two variables: ‘the characteristics of cholesteatoma’ and ‘the surgical procedure’. In the Netherlands, ENT surgeons often divide surgical procedures of the ear into two main groups: canal wall up mastoidectomy (CWU) and canal wall down mastoidectomy (CWD). In recent years, techniques like mastoid obliteration (MO) have been added to these procedures [3]. In the CWU procedure, the posterior bony ear canal is left intact keeping the ear canal and mastoid cavity separated from each other. During the CWD procedure, the posterior bony ear canal is removed and an open cavity remains. Obliteration can be combined with both procedures. It prevents the tympanic membrane from retracting towards the cavity, since there is no space to retract to [3].

In practice, there is a great variation in surgical techniques used by ENT surgeons and a division into just ‘canal wall up’ and ‘canal wall down’ hardly covers these. The CWU/CWD classification has been suggested in 1985 and adjusted in 1993 [4, 5]. Many possible classifications and modifications have been proposed since and all were recently reviewed [2]. None of these classifications have been internationally accepted. To overcome this problem, the International Otology Outcome Group (IOOG) recently proposed a new classification system, with the acronym SAMEO-ATO. This classification was developed according to an international Delphi consensus method. It especially focuses on a system to categorize mastoid bone operations (SAMEO) and middle ear surgeries (ATO). The acronym stands for each subgroup of surgeries of the mastoid and middle ear namely; stage of operation, approach, mastoidectomy procedure, external auditory canal reconstruction, obliteration of mastoid cavity, access, tympanic membrane (TM) repair, ossicular chain repair and is further explained in Supplemental Digital Content 1, which demonstrates the SAMEO-ATO framework [6]. This framework could be a very useful tool in categorizing the mastoid and middle ear surgeries, due to the schematic framework and very helpful drawings of the different options per procedure with explanatory text.

The aim of this study was to assess the newly proposed classification system, by means of retrospectively comparing the M-stage of the SAMEO-ATO classification to the more common CWU and CWD procedures (with or without mastoid obliteration techniques) as used in the Dutch prospective cholesteatoma study. In addition, surgical reports were classified to the other subgroups of the SAMEO-ATO classification.

## Methods

### Patients

The Dutch Cholesteatoma Data (DCD) is a nationwide multicenter study in the Netherlands. Sixteen secondary and tertiary care hospitals, spread across the Netherlands participate in this study. Its aim is to build a national database of adult patients who were operated for cholesteatoma. From March 2017 to March 2018, 135 patients from thirteen centers were included. Three centers did not include patients due to organizational difficulties. Inclusion criteria were: (a) 18 years of age or older; (b) good Dutch language proficiency; (c) surgery for eradication of primary, acquired or recurrent cholesteatoma. An exclusion criteria was: (a) pregnancy. After inclusion, patients were de-identified and a number code was provided.

This study was approved by the Medical Ethical Committee of the VU University Medical Center, Amsterdam (Reference Number NL50862.029.16). Written informed consent was obtained prior to study participation.

### Procedure

A clinical research file (CRF) was created in which the ENT surgeons from the participating centers were asked to register demographic, surgical, post-op examination and follow-up data from their own included patients. These CRFs were added to the national cholesteatoma database created in Castor EDC https://www.castoredc.com. The ENT surgeons had to classify their surgical procedures as ‘CWU’, ‘CWD’, ‘CWU with MO’, ‘CWD with MO’, or ‘subtotal petrosectomy (SP)’. In addition, screenshots of the surgical reports without patient information were added to the CRF.

Registration of the classification of the performed surgical procedure was verified with the surgical report. When registration in the CRF differed from the surgical report, the assigned ENT surgeon was consulted to check for possible registration errors.

All surgical reports were analyzed and retrospectively classified according to the SAMEO-ATO classification by two independent ENT researchers (PM, SA), using the SAMEO-ATO framework [7] (https://www.ioog.net). If it was not possible to classify a specific subgroup of the classification, this was marked with a question mark. An exception to this was made for the ‘mastoid bone extirpation’ (M-stage). In some cases, the described used techniques in the surgical reports were difficult to fit to the M-stage of the SAMEO-ATO classification (such as lateral epitympanotomy and anterior tympanotomy). In these cases, the assigned ENT surgeon was asked which M-stage corresponded best with the specific procedure that was performed. The M-stage is divided into ten stages: Mx (no mastoidectomy), M1a (canal wall preserved), M1b (canal wall preserved and posterior tympanotomy), M2a (only scutum removed), M2b (scutum removed and postero-superior canal wall removed), M2c (whole canal removed), M3a (subtotal petrosectomy with preservation of the otic capsula, including exenteration of all mastoid and middle ear pneumatized cells), M3b (subtotal petrosectomy with removal of the otic capsula, including labyrinthectomy and/or removal of the cochlea) and the combinations M1a + 2a and M1b + 2a.

### Statistical analyses

Cross tabulations were made in order to examine the relationship between the categorical variables mastoid bone extirpation (M-stage) according to the SAMEO-ATO classification and the surgical procedure classified according to the classification system used in the study (CWU, CWU + MO, CWD, CWD + MO or SP). For statistical analysis, the CWU group consists of both CWU and CWU + MO. And CWD consists of both CWD and CWD + MO. Column proportion tests (or *Z* tests) and Chi-square tests were performed in order to determine a possible correlation between the SAMEO-ATO classification and classification as used in the study. In the event that the expected count in a M-stage group was less than 5, a Fisher’s exact test was performed. Statistical significance was set at *P* < 0.05. Statistical analysis was carried out using SPSS 24.

## Results

**Fig. 1:**
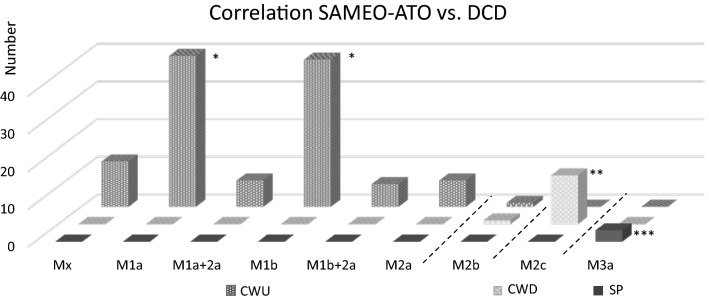
Correlation between SAMEO-ATO classification and CWU/CWD classification as used by ENT surgeon. On the *z*-axis the CWU(+MO) = canal wall up with or without mastoid obliteration is depicted in spotted dark-grey, CWD(+MO) = canal wall down with or without mastoid obliteration depicted in spotted light-grey and SP = subtotal petrosectomy depicted in black. Symbol (*) indicates a significant correlation with CWU. Symbol (**) indicates a significant correlation with CWD and symbol (***) indicates a significant correlation with SP. On the *y*-axis the number of the used M-stages are depicted

The registration in the CRF did not match the surgical report in thirteen cases and the CRF was subsequently changed after re-evaluation by the ENT researchers in agreement with the assigned ENT surgeon. Six cholesteatoma patients had a surgical report that was incomplete and hence could not be used for analyses. Therefore, these patients were excluded. In total, 129 surgical reports from 129 patients originating from 13 hospitals were included and were analyzed and classified by two ENT researchers. Table 1 and Fig. 1 show the number of the different M-stages found in this cohort correlating with either CWU, CWD or SP.

**Table 1:** M-stages versus either CWU, CWD or SP

M-stages	Number (*N*) and percentage (%)		
	CWU	CWD	SP
Mx (no mastoidectomy)	12 (100%)	–	–
M1a (canal wall preserved)	40 (100%)	–	–
M1b (canal wall preserved and posterior tympanotomy)	39 (100%)	–	–
M2a (only scutum removed)	7 (100%)	–	–
M2b (scutum + postero-superior wall removed)	1 (50%)	1 (50%)	–
M2c (mastoidectomy with whole canal wall removed)	–	13 (100%)	–
M3a (otic capsule preserved)	–	–	3 (100%)
M3b (otic capsule removed)	–	–	–
Combination M1a + 2a	7 (100%)	–	–
Combination M1b + 2a	6 (100%)	–	–
Total	112 (86.8%)	14 (10.9%)	3 (2.3%)

Count and the percentage of the total number of performed CWU, CWD or SP within the M classification are given

As shown in Table 1 (Fig. 1), Mx, M1a, M2a, M1b, M1a + 2a, and M1b + 2a procedures were only performed in reports classified as CWU. A M2c procedure was classified as CWD in all cases. However for a M2b procedure, there was no preferred choice for either the CWU or CWD procedure. A M3a procedure was only described in SP and a M3b procedure was not reported in this cohort. *Z* test for correlation between the mastoid bone extirpation (M) and CWU procedure is significant for M1a and M1b procedure. *Z* test for correlation between the mastoid bone extirpation (M) and CWD procedure was significant for M2c. The Chi-Square tests performed for both correlations of CWU and CWD with the M-stages were statistical significant. However, due to a M-stage with an expected count less than 5 (M2b procedure), a two tailed Fisher exact test was performed. These performed Fisher exact tests were both significant: CWU (*P* < 0.001) and CWD (*P* < 0.001).

Next to correlating the M-stage with the currently used classifications of the surgical procedures, surgical reports were also scored for the other subgroups of the classification. In 95 surgical reports it was possible to also score the other subgroups of the SAMEO-ATO classification. However, in 34 surgical reports it was not possible to classify every part of the SAMEO-ATO classification. Of these 34, two surgical reports were not clear on how external bony wall repair (E) was performed, 22 times it was not possible to classify the access to the middle ear (A), five times the tympanic membrane reconstruction was not clear (T), seven times the ossicular reconstruction was not properly described (O) and in two surgical reports there were multiple elements that could not be classified.

## Discussion

This study was performed to investigate the correlation between the M-stage of the newly proposed SAMEO-ATO classification and the better known CWU and CWD classification. The data in Fig. 1 show the correlation between the M-stages (mastoid bone extirpation) of the SAMEO-ATO classification and classification (CWU, CWD with or without obliteration) as used in the multicenter study. There seems a clear correlation between almost all M-stages and the former CWU or CWD classification. The CWU procedures correlate with Mx (no mastoidectomy), M1a (canal wall preserved), M1b (canal wall preserved and posterior tympanotomy), M2a (only scutum removed) and the combinations M1a + 2a, M1b + 2a. Especially, the correlation of M2a (atticotomy/scutum removal) with CWU is interesting. M2 procedures are all surgeries with some degree of posterior wall removal. M2a is the most mild form of these surgeries and is considered by the (Dutch) ENT surgeons a widening of the surgical view but still a CWU procedure. The *Z *test performed for correlation between the M-stage and CWU was only significant for M1a and M1b. This means that whenever a CWU procedure is performed, this is significantly more often a classified as an M1a or M1b procedure than when a CWD procedure is performed. M2b (mastoidectomy with superior scutum and postero-superior canal wall removed) was scored in only two cases. In one of them, the surgical report was classified as CWU and in the other as CWD. This procedure may be considered in between CWU or CWD and therefore be not classifiable in the old system. A caveat however is the low number of cases in this series, so a definite conclusion cannot be drawn. The interpretation of the experienced ENT surgeons, the low number of use and the in-between status of leaving the ear canal intact or taking it away may indicate that M2b is regarded as an unfavorable surgical procedure. Of course there is no scientific evidence to support this. M2c (mastoidectomy with whole canal wall removed) is only described in CWD procedure and the *Z* test for correlation between this M-stage and CWD was significant. This means that when a CWD procedure is performed, this is significantly more often classified as an M2c procedure than when a CWU procedure is performed. The M3a procedure (SP; otic capsule preserved) was only described in SP. *Z* tests performed were not significant for Mx, M1a + 2a, M1b + 2a and M2a procedure when analyzing CWU procedures, none of the aforementioned procedures described in the surgical reports were classified as CWD. In a larger series with higher numbers of these procedures, a significant correlation may be found.

Retrospectively applying the complete SAMEO-ATO classification to the surgical reports was not (fully) possible in 34 cases, in which the majority was due to the classification of the middle ear procedure (ATO). In the user notes, the IOOG advises to score all subgroups of the SAMEO-ATO and not only one complete subgroup. These 34 incomplete surgical reports could be due to the retrospective application of the SAMEO-ATO classification. The IOOG recognizes in the user notes and publication that the SAMEO-ATO is established for prospective use and that retrospective use may be impaired due to incomplete previously collected data [7]. Especially the precise description of the access to middle ear (A) was often not clearly described in the surgical report. Terms as ‘widening the external ear canal’ or ‘drilling the external ear canal’ were used often, making it difficult to differentiate between A1 (widening of the posterior portion of tympanic sulcus, including canal curettage or drilling to visualize the ossicular chain or hypotympanum) and A2 (partial or circumferential widening of the bony canal/canalplasty). Although surgeons seem to find canalplasty an important surgical step (this procedure was performed in many cases), precise description was considered less relevant for the surgical report. In the user notes a section is incorporated related to the access to middle ear (A). However, this user notes section only describes the distinction between A2 and A3. We advise to incorporate in the user notes a section that describes the distinction between A1 and A2. Problems in classifying the ossicular chain (O) mostly occurred in recurrent or residual cholesteatomas. Ossicular chain reconstruction was performed during previous surgery, but the type of reconstruction was not mentioned in the surgical report, which made it impossible to classify. Therefore, we recommend to incorporate an extra acronym, for example ‘Or’ that represents revision surgery in the ossicular chain reconstruction section of the user notes, such as the S2r for stage of surgery.

There were some limitations of the used methods in this study. It is important to understand that the multicenter database consists of a small amount of cholesteatoma patients compared to the estimated 1700 patients that are treated annually in the Netherlands. These data can only give a first impression of how cholesteatoma is treated in the Netherlands. In order to get a more veracious view on the surgical procedures performed in the Netherlands, it is necessary to include and analyze all patients operated because of cholesteatoma during one year in future research. The newly proposed SAMEO-ATO classification is much more detailed in the registration of surgical procedures than ENT surgeons currently are used to. By applying the classification retrospectively instead of the intended prospective manner, not all surgical reports could be fully classified. This limitation is also mentioned in the user notes of the SAMEO-ATO. The IOOG group advises to allocate the performed procedure to the closest fit and register the details of differences separately. In this way, data can be generated that may stimulate new updates of the SAMEO-ATO classification.

Another limitation was the registration in the CRF which led to thirteen cases that had to be re-classified, in agreement with the assigned ENT surgeon, because of a mismatch with the surgical notes. A CRF and ideally an electronic patient file should register or suggest automatically from the surgical report a classification to register. Human error could be diminished when computer assisted registration is performed.

Some of the included surgical reports in this study described a revision surgery. To distinguish between a primary and revision surgery when using the SAMEO-ATO, a few points must be taken into account. First of all, when performing a primary surgery the SAMEO-ATO must be filled in completely. When describing a revision surgery, the information about the previous surgery performed scored by means of the SAMEO-ATO must be leading. Therefore, only if the situation in the middle ear has changed, new information scored with the SAMEO-ATO must be described in the surgical report. Secondly, if the situation did not change, no new information must be registered in the surgical report, or old info must be quoted as unchanged. SAMEO ATO is a description of the surgical procedure, so if the chain reconstruction was done in the previous surgery this should not be stated as a new procedure. SAMEO procedure could therefore in some cases be written down without the ATO procedure. However, to register both new and old info in the surgical report may serve as a reference book. Otherwise the surgeon must read older surgical reports to be able to interpret the last surgical report.

To improve implementation of the SAMEO-ATO in daily practice some additions in registration may be necessary. The user notes state that the SAMEO-ATO classification is meant to categorize surgical procedures but not to register the impact of a disease. Since uniform registration to aid comparison of surgical outcomes is the aim of the SAMEO-ATO, registering disease characteristics and outcomes are important too. Disease characteristics (the pathology) can be registered by means of the STAMCO classification for example [8]. Classification of both surgical outcomes by means of the SAMEO-ATO and the classification of the pathology should always be performed together. It is important to register data uniformly in order to improve the evidence base, compare outcomes, enhance quality and form guidelines [9, 10]. The SAMEO-ATO is a detailed surgical classification system and this detail enhances the amount of information collected, is specific in describing the subgroups and therefore could contribute to achieve the goals of registering uniformly. The accompanying SAMEO-ATO framework of the different options of the subgroups is a very helpful tool in the categorization process. Drawings are well-defined and the additional information is clear.

### Future perspectives

Due to the schematic overview in the registration of the surgical procedure, the SAMEO-ATO seems to be a good classification for uniform registration. However, the classification should evolve over time as it has many details which make it less user friendly. In addition, we have proposed some suggestions for the User Notes that accompany the classification presented on https://www.ioog.net and https://sameo-ato.surgery, to improve the use of the SAMEO-ATO. Although it is necessary to make some modifications to the SAMEO-ATO classification to make it less user-friendly, we encourage to use this system to internationally start registering uniformly.

## Conclusion

An international accepted classification system for uniform registration of the surgical procedure is necessary to allow ENT surgeons to present their results and contribute to the development of better evidence and guidelines. SAMEO-ATO will be very beneficial in that manner and is much more detailed than the common CWU or CWD classification. Classification of the Mastoid bone extirpation is well comparable to the used CWU/CWD system but future research must give more insight on the comparability and usability of the M2b stage. Furthermore, this classification will hopefully become more familiar among ENT surgeons world-wide, and will evolve over time to become best practice and more user friendly.

## Electronic supplementary material

Below is the link to the electronic supplementary material. Fig. 1 Summary of the IOOG SAMEO-ATO framework. Used with permission of the author and journal of international advanced otology [6].

Supplementary file1 (PDF 2815 kb)

Supplementary file2 (PDF 68 kb)

## Data Availability

Data is available when requested.

## References

[1] Rutkowska J, Ozgirgin N, Olszewska E (2017). Cholesteatoma definition and classification: a literature review. J Int Adv Otol.

[2] Merkus P, Kemp P, Ziylan F, Yung M (2018). Classifications of mastoid and middle ear surgery: a scoping review. J Int Adv Otol.

[3] Van Der Toom HFE, Van Der Schroeff MP, Pauw RJ (2018). Single-stage mastoid obliteration in cholesteatoma surgery and recurrent and residual disease rates a systematic review. JAMA Otolaryngol Head Neck Surg.

[4] Marres E (1985) Management of the mastoid—the UDT-system; a new classification in ear surgery. In: Surgery and pathology of the middle ear. Springer, Dordrecht, pp 58–61

[5] Tos M (1993). Manual of middle ear surgery.

[6] Yung M, James A, Merkus P (2018). International otology outcome group and the international consensus on the categorization of tympanomastoid surgery. J Int Adv Otol.

[7] Yung M, James A, Merkus P et al (2018) User guide for the SAMEO-ATO categorisation of tympanomastoid surgery. J Int Adv Otol 14(2):216–226. 10.5152/iao.2018.5553. https://www.ioog.net 10.5152/iao.2018.5553 PMC6354466 30100547

[8] Merkus P, Tije FA, Stam M, Tan FML, Pauw RJ (2017). Implementation of the “EAONO/JOS definitions and classification of middle ear cholesteatoma” from STAM to STAMCO. J Int Adv Otol.

[9] Mandavia R, Knight A, Carter AW (2018). What are the requirements for developing a successful national registry of auditory implants? A qualitative study. BMJ Open.

[10] Jones B, Vaux E, Olsson-Brown A (2019). How to get started in quality improvement. BMJ.

