# Supplementary material for: Evaluation of the SAMEO-ATO surgical classification in a Dutch cohort
Source: Eur Arch Otorhinolaryngol. 2020 Jun 11;278(3):653–8. doi: 10.1007/s00405-020-06109-1 (PMC7895777; doi:10.1007/s00405-020-06109-1)
Supplement: Supplementary file 1 — Supplementary file1 (PDF 2815 kb) [file 405_2020_6109_MOESM1_ESM.pdf]

Summary of IOOG SAMEO-ATO framework

www.IOOG.net

S Stage of surgery

**S1**	Primary (first surgery)
**S_{2p}**	Planned (2nd look or staged procedure)
**S_{2r}**	Revision (unplanned)

A Approach

**A1**	Endoscopic transcanal
**A2**	Microscopic transcanal
**A3**	Endaural
**A4**	Retroauricular

M Mastoidectomy

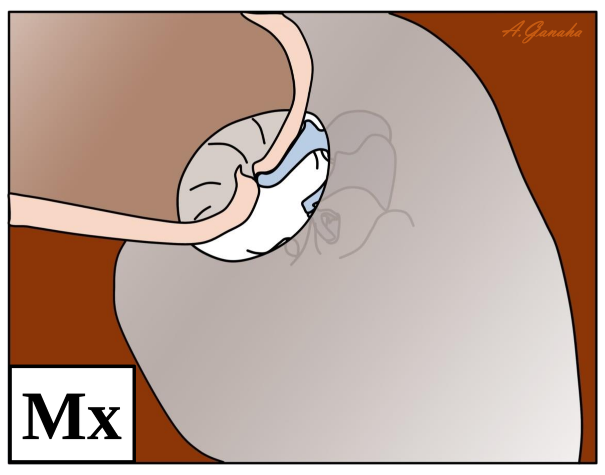

no mastoidectomy

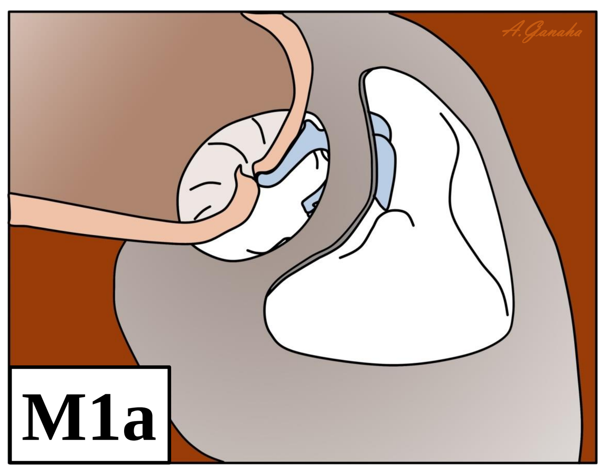

canal wall preserved

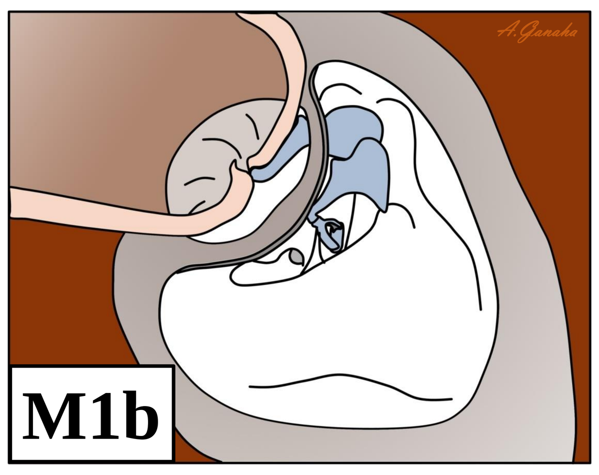

+ posterior tympanotomy

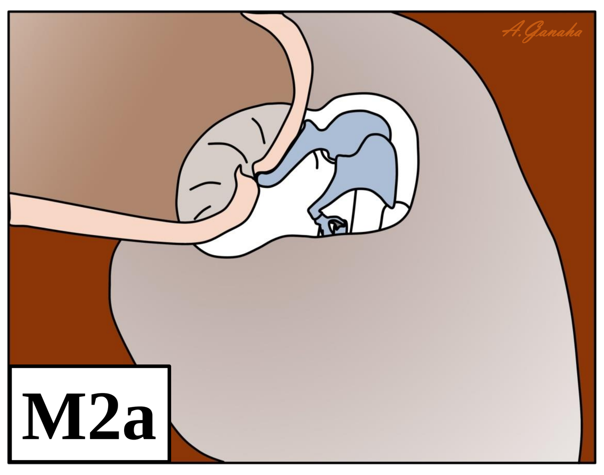

only scutum removed

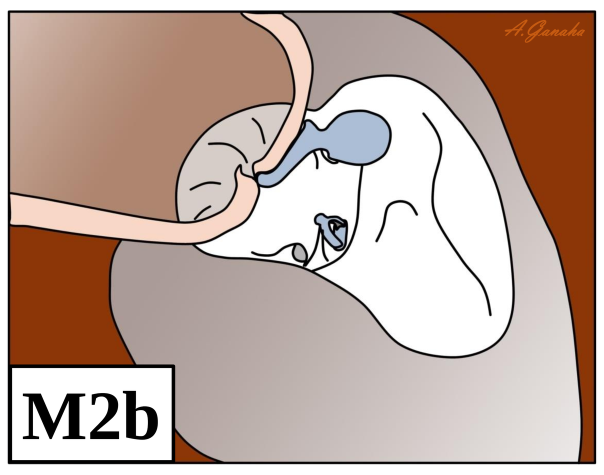

scutum + postero-superior wall removed

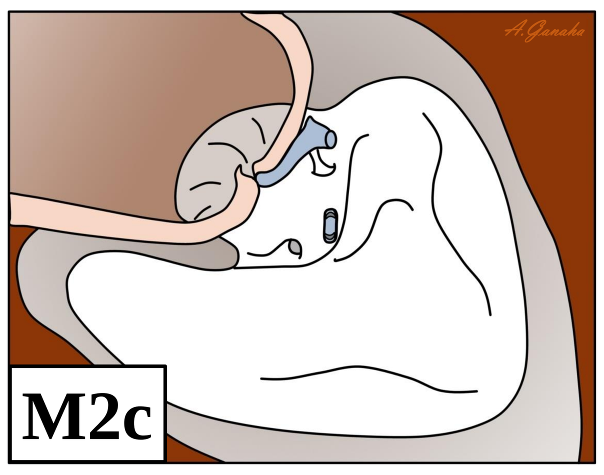

whole canal wall removed

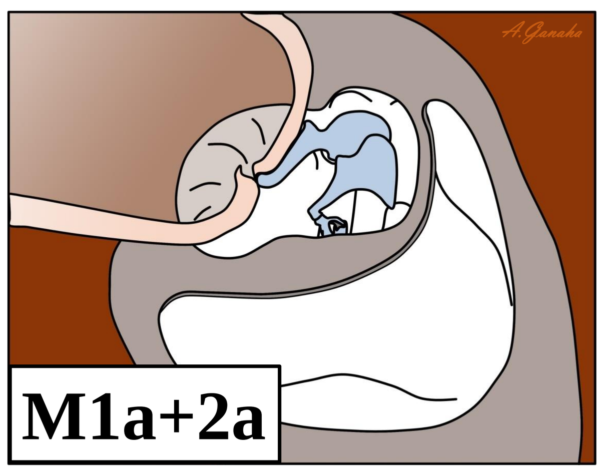

combination of M_{1a} and M_{2a}

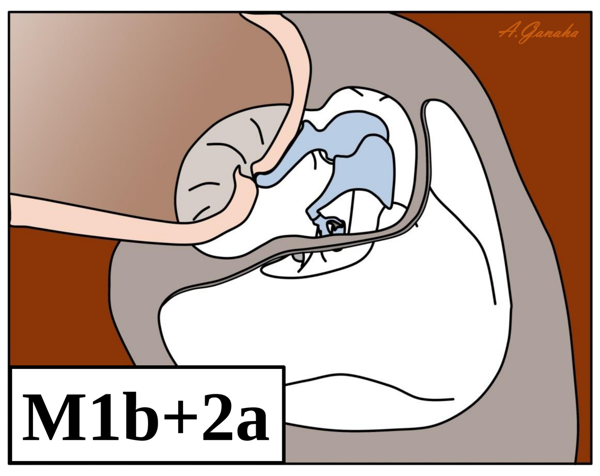

combination of M_{1b} and M_{2a}

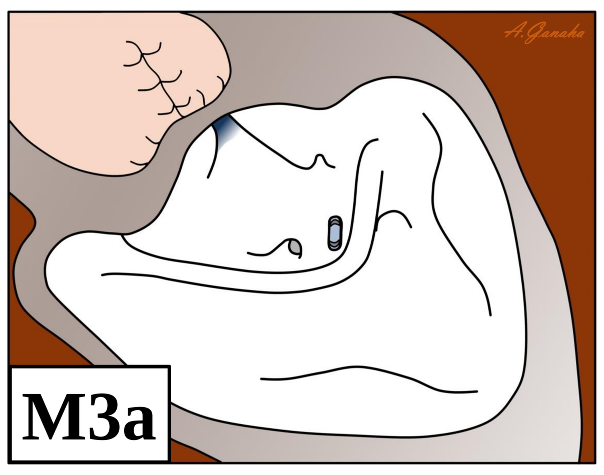

subtotal petrosectomy; otic capsula preserved

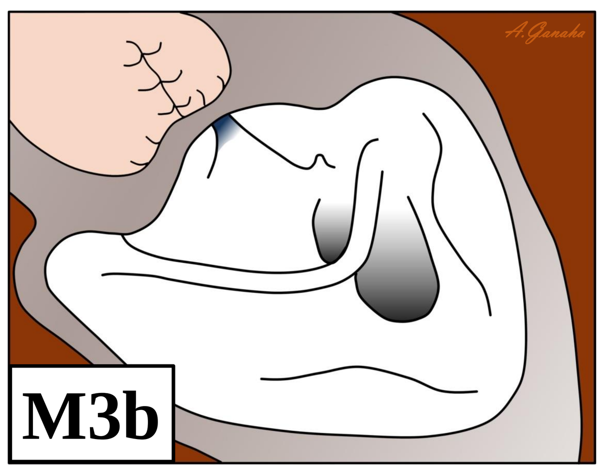

subtotal petrosectomy; otic capsula removed

E External ear canal reconstruction

**Ex**	No external ear canal reconstruction
**E1**	Reconstruction with soft materials†
**E2**	Reconstruction with rigid materials†

† *space behind graft not obliterated*

O Obliteration of mastoid cavity

**Ox**	No obliteration
**O1**	Partial obliteration
**O2**	Total obliteration

A Access to middle ear

**Ax**	No bone removal from the external ear canal wall (flattening of suture line alone is still considered as Ax)
**A1**	Widening of the posterior portion of tympanic sulcus (including canal curettage or drilling to visualise the ossicular chain or hypotympanum)
**A2**	Partial or circumferential widening of the bony canal (canalplasty)
**A3**	Total canalplasty with soft tissue grafting of exposed bone

T Tympanic membrane

**Tx**	No tympanic membrane grafting performed
**Tn**	Original tympanic membrane preserved
**T1**	Supplement to intact tympanic membrane (reinforcement)
**T2**	Partial tympanic membrane grafting
**T3**	Subtotal / total tympanic membrane grafting

O Ossicular chain

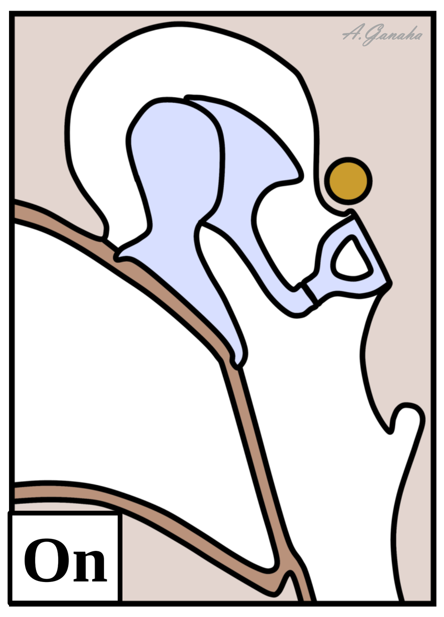

intact chain preservation

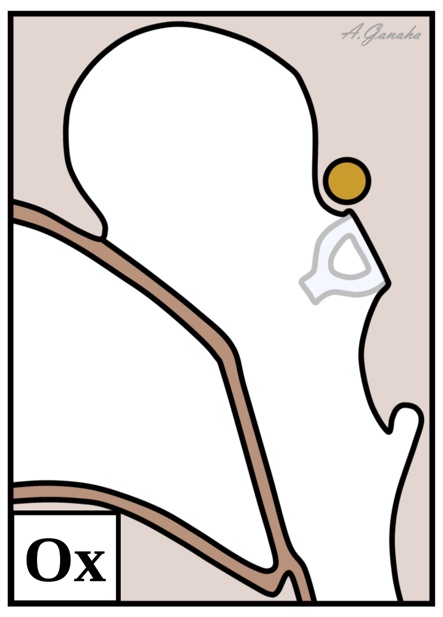

no reconstruction done

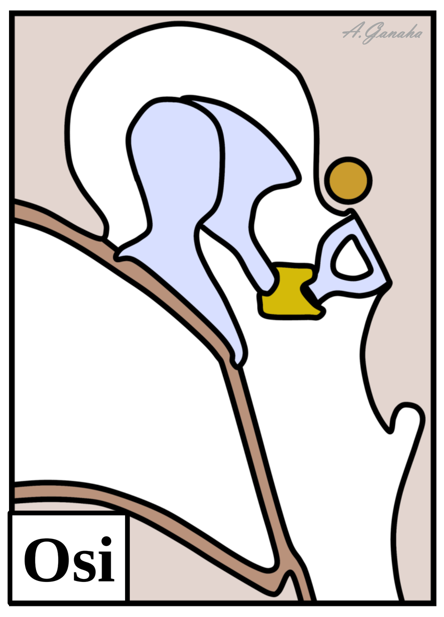

incus to stapes

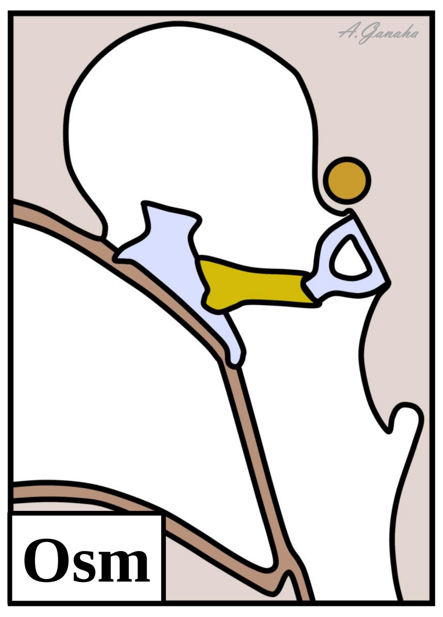

malleus to stapes

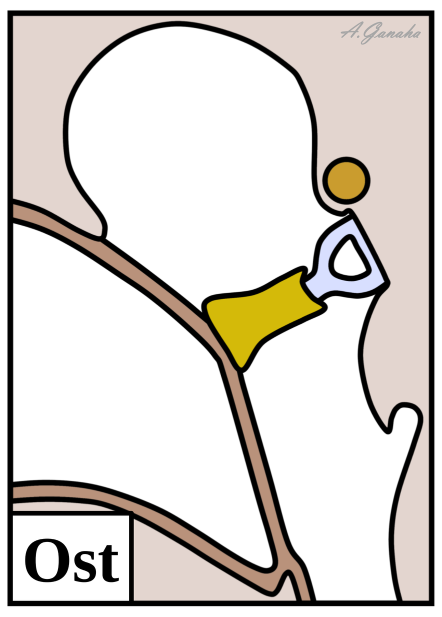

TM to stapes

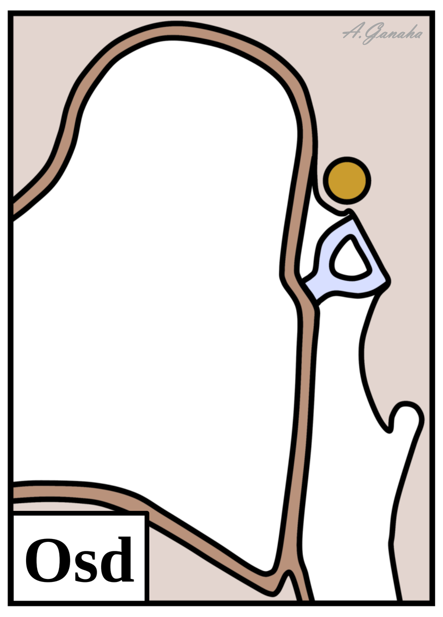

TM directly on stapes

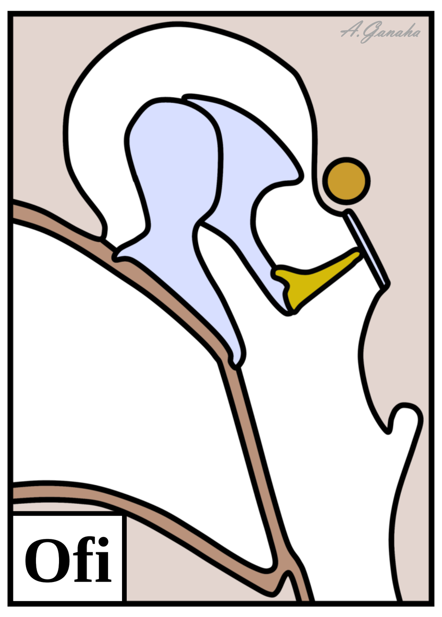

incus to footplate

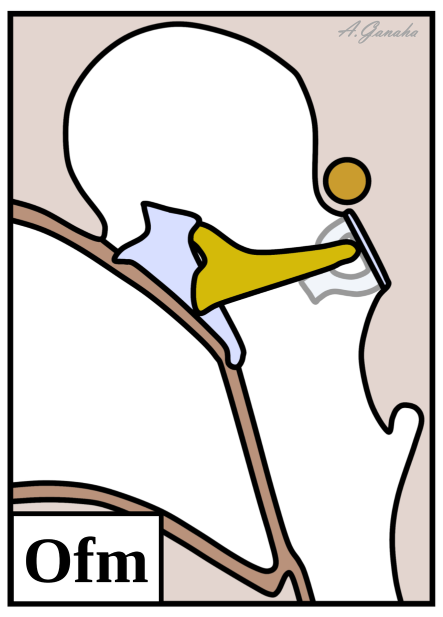

malleus to footplate

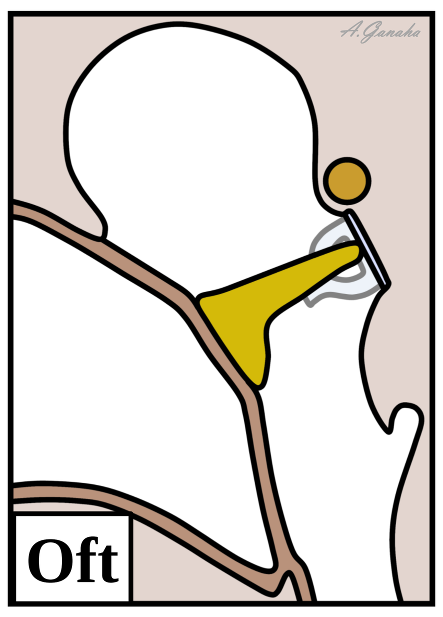

TM to footplate

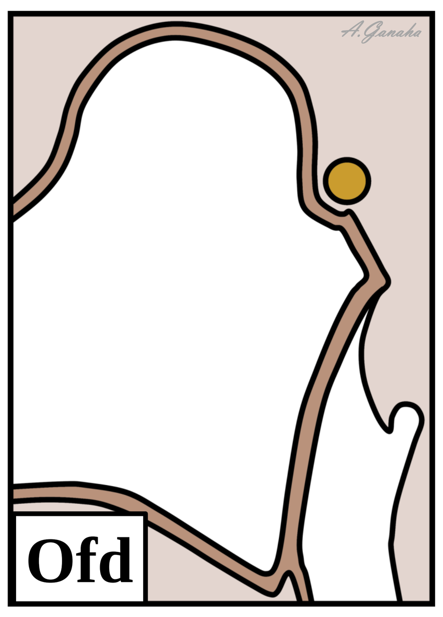

TM directly on footplate

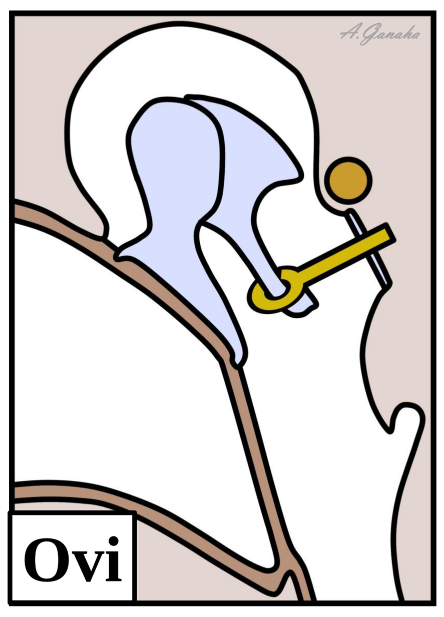

incus to vestibule

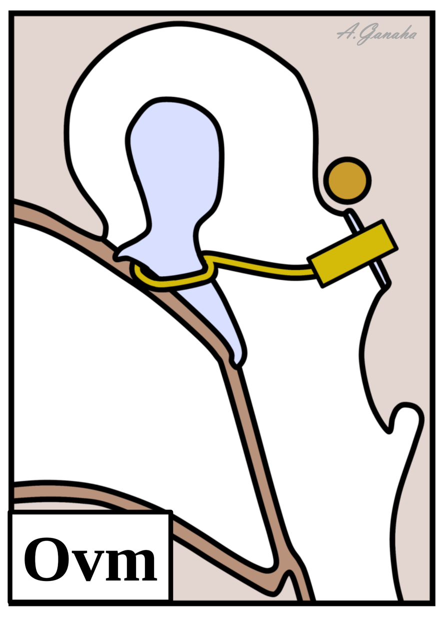

malleus to vestibule

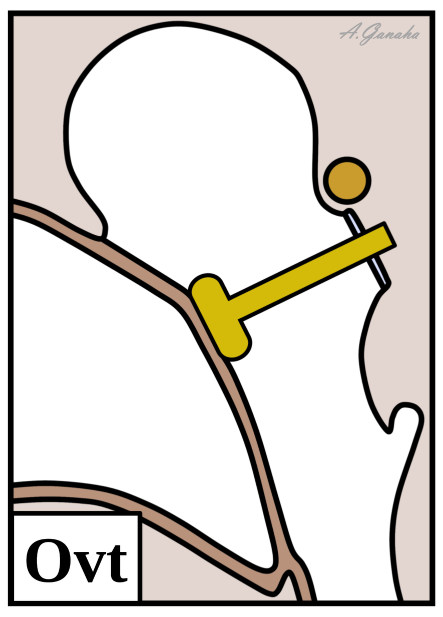

TM to vestibule
