# Supplementary material for: Evaluation of the SAMEO-ATO surgical classification in a Dutch cohort
Source: Eur Arch Otorhinolaryngol. 2020 Jun 11;278(3):653–8. doi: 10.1007/s00405-020-06109-1 (PMC7895777; doi:10.1007/s00405-020-06109-1)
Supplement: Supplementary file 2 — Supplementary file2 (PDF 68 kb) [file 405_2020_6109_MOESM2_ESM.pdf]

Heike J. **Nyst** MD; Department of Otolaryngology–Head and Neck Surgery, Onze Lieve Vrouwe Gasthuis, Amsterdam
Annemarie P. **Graveland** MD PHD; Department of Otolaryngology–Head and Neck Surgery, Noordwest Ziekenhuisgroep, Alkmaar & Den Helder
Gerben G. **Kingma** MD; Department of Otolaryngology–Head and Neck Surgery, Noordwest Ziekenhuisgroep, Alkmaar & Den Helder
Jeroen W.L. **van Lange** MD; Department of Otolaryngology–Head and Neck Surgery, Noordwest Ziekenhuisgroep, Alkmaar & Den Helder
Guido T.M. **de Kuyper** MD; Department of Otolaryngology–Head and Neck Surgery, Noordwest Ziekenhuisgroep, Alkmaar & Den Helder
Johan M. **Schmidt** MD; Department of Otolaryngology–Head and Neck Surgery, Noordwest Ziekenhuisgroep, Alkmaar & Den Helder
Jantine **Venker** MD; Department of Otolaryngology–Head and Neck Surgery, Noordwest Ziekenhuisgroep, Alkmaar & Den Helder
Dick J. **Warmerdam** MD PHD; Department of Otolaryngology–Head and Neck Surgery, Noordwest Ziekenhuisgroep, Alkmaar & Den Helder
Tjasse D. **Bruintjes** MD PHD; Department of Otolaryngology–Head and Neck Surgery, Gelre ziekenhuizen, Apeldoorn & Zutphen
Raphael J.B. **Hemler** MD PHD; Department of Otolaryngology–Head and Neck Surgery, Gelre ziekenhuizen, Apeldoorn & Zutphen
Kees J. **Langenhuijsen** MD; Department of Otolaryngology–Head and Neck Surgery, Gelre ziekenhuizen, Apeldoorn & Zutphen
Stephanie M. **Winters** MD PHD; Department of Otolaryngology–Head and Neck Surgery, Gelre ziekenhuizen, Apeldoorn & Zutphen
Jeroen **Mud** MD; Department of Otolaryngology–Head and Neck Surgery, Gelre ziekenhuizen, Apeldoorn & Zutphen
Adriaan F. **Holm** MD PHD; Department of Otolaryngology–Head and Neck Surgery, Wilhelmina ziekenhuis, Assen
Ferdinand A.W. **Peek** MD; Department of Otolaryngology–Head and Neck Surgery, Reinier de Graaf Groep, Delft
Henk M. **Blom** MD PHD; Department of Otolaryngology–Head and Neck Surgery, Haga ziekenhuis, Den Haag
Steven J.H. **Bom** MD PHD; Department of Otolaryngology–Head and Neck Surgery, Deventer Ziekenhuis, Deventer
Henri-Jacques **Tjong-Ayong** MD; Department of Otolaryngology–Head and Neck Surgery, Nij Smellinghe Ziekenhuis, Drachten
Jan Pieter **de Mönnink** MD; Department of Otolaryngology–Head and Neck Surgery, Sint-Anna Ziekenhuis, Geldrop
Nynke **Boelstra-van Cruijsen** MD PHD; Department of Otolaryngology–Head and Neck Surgery, Martini ziekenhuis, Groningen
Jurjan R. **de Boer** MD PHD; Department of Otolaryngology–Head and Neck Surgery, Martini ziekenhuis, Groningen
Sietske F. **Meinesz** MD; Department of Otolaryngology–Head and Neck Surgery, Haags Medisch Centrum, Den Haag
Josephina M. **Kruyt** MD; Department of Otolaryngology–Head and Neck Surgery, Haags Medisch

Centrum, Den Haag
David R. **Colnot** MD PHD; Department of Otolaryngology–Head and Neck Surgery, Diakonessenhuis, Utrecht
Jasper J. **Quak** MD PHD; Department of Otolaryngology–Head and Neck Surgery, Diakonessenhuis, Utrecht
Pepijn A. **Borggreven** MD PHD; Department of Otolaryngology–Head and Neck Surgery, Diakonessenhuis, Utrecht
Rick **van de Langenberg** MD PHD; Department of Otolaryngology–Head and Neck Surgery, Diakonessenhuis, Utrecht
Adriana J.G.E. **Wedler-Peeters** MD; Department of Otolaryngology–Head and Neck Surgery, Zuyderland MC, Sittard-Geleen & Heerlen
Jorien **Snel-Bongers** MD PHD; Department of Otolaryngology–Head and Neck Surgery, Zuyderland MC, Sittard-Geleen & Heerlen
Eelco E. **Kummer** MD; Department of Otolaryngology–Head and Neck Surgery, Zaans Medisch Centrum, Zaandam
Annette J. **ter Schiphorst** MD; Department of Otolaryngology–Head and Neck Surgery, Isala ziekenhuis, Zwolle
